# Thank you to our contributors: reflections on 2024

**DOI:** 10.18849/ve.v10i1.718

**Published:** 2025-01-31

**Authors:** Peter Cockcroft

**Keywords:** thank you, peer-review, reviewers, contributors, editorial process, indexing

## Abstract

All submissions to *Veterinary Evidence* are handled by an Associate Editor specialising in the relevant topic area. We would like to thank these volunteer veterinary professionals for their expertise and time to support the journal. Associate Editors play a central role, assessing submitted papers, guiding the peer review process, and making decision recommendations based on peer review reports. We have welcomed six new Associate Editors to the journal in recent months, and we welcome new applications to join our editorial team. Our grateful thanks also extend to our peer reviewers, who are critical in ensuring we maintain the highest standards of excellence in our published articles.

In conjunction with our Editorial Board and publishing team, we have developed a new strategy for *Veterinary Evidence*, aiming to increase the visibility and findability of journal content and develop ways to measure real impact. We will aim to increase the volume and breadth of what we publish and expand the journal’s editorial capacity and capability to enable that. Quality remains central and we will continue to set strong standards for published content through our policies and processes, and to ensure we make the best use of the support from RCVS Knowledge, we will continually improve editorial processes and timelines to increase editorial efficiency and reduce publication timelines.

We are pleased to announce that *Veterinary Evidence* has recently been accepted for indexing in Scopus and has also been approved for inclusion in PubMed Central. The technical work for indexing to happen is underway, and we will continue to explore options to increase journal content visibility.

Knowledge Summaries guide readers through the evidence on a clinical question. To help with understanding the context of an included study, we have expanded the evidence summary sections to include the full title of the study, a link out to the original article, and a summary of the main aims. The journal continues to grow annually in terms of users and views, and in 2024 we saw a 13% increase in users and a 5% in page views. Submission to the journal also continue at a steady rate.

The process for updating published Knowledge Summaries has now been agreed. The journal runs an annual report of Knowledge Summaries that have search dates (the date that the search was carried out) that are 3 to 4 years old. For that set of papers, we review the usage metrics (views, downloads, citations) and consider whether the questions remain clinically relevant. On this basis, the Editor in Chief will select the top five papers of interest under each of the main three species categories (small animal, production animal, and equine), and we will aim to publish updates of these knowledge summaries.

Finally, we would like to encourage the continued submission of clinical queries and papers for consideration for publication so that the journal can continue to thrive and go from strength to strength.

Professor Peter Cockcroft

Editor in Chief, *Veterinary Evidence*

## Abstract

All submissions to Veterinary Evidence are handled by an Associate Editor specialising in the relevant topic area. We would like to thank these volunteer veterinary professionals for their expertise and time to support the journal. Associate Editors play a central role, assessing submitted papers, guiding the peer review process, and making decision recommendations based on peer review reports. We have welcomed six new Associate Editors to the journal in recent months, and we welcome new applications to join our editorial team. Our grateful thanks also extend to our peer reviewers, who are critical in ensuring we maintain the highest standards of excellence in our published articles.

In conjunction with our Editorial Board and publishing team, we have developed a new strategy for *Veterinary Evidence*, aiming to increase the visibility and findability of journal content and develop ways to measure real impact. We will aim to increase the volume and breadth of what we publish and expand the journal’s editorial capacity and capability to enable that. Quality remains central and we will continue to set strong standards for published content through our policies and processes, and to ensure we make the best use of the support from RCVS Knowledge, we will continually improve editorial processes and timelines to increase editorial efficiency and reduce publication timelines.

We are pleased to announce that *Veterinary Evidence* has recently been accepted for indexing in Scopus and has also been approved for inclusion in PubMed Central. The technical work for indexing to happen is underway, and we will continue to explore options to increase journal content visibility.

Knowledge Summaries guide readers through the evidence on a clinical question. To help with understanding the context of an included study, we have expanded the evidence summary sections to include the full title of the study, a link out to the original article, and a summary of the main aims. The journal continues to grow annually in terms of users and views, and in 2024 we saw a 13% increase in users and a 5% in page views. Submission to the journal also continue at a steady rate.

The process for updating published Knowledge Summaries has now been agreed. The journal runs an annual report of Knowledge Summaries that have search dates (the date that the search was carried out) that are 3 to 4 years old. For that set of papers, we review the usage metrics (views, downloads, citations) and consider whether the questions remain clinically relevant. On this basis, the Editor in Chief will select the top five papers of interest under each of the main three species categories (small animal, production animal, and equine), and we will aim to publish updates of these knowledge summaries.

Finally, we would like to encourage the continued submission of clinical queries and papers for consideration for publication so that the journal can continue to thrive and go from strength to strength.

Professor Peter Cockcroft 

Editor in Chief, *Veterinary Evidence*

### Peer reviewers in 2024 :

Jane Alexander

Fergus Allerton

Anthi Anatolitou

Máirín-Rua Ní Aodha

Anant Andy Banerjee

Duncan Barnes

Rachel Basa

Andrew Bell

Luca Bellini

Rebecca Bhalla

Jocelyn Bisson

Cameron Black

Nicola Blackie

Adrian Boswood

Sarah Bouyssou

Camilla Brocklehurst

Louise Buckley

John Campbell

Tom Candy

James Carmalt

Simbarashe Chipunga

Jake Chitty

John Chitty

Louise Clark

Ana Cloquell Miro

Gwen Covey-Crump

Amanda Curtis

Niranjala De Silva

Zoe Demery

Isabelle Desprez

Dave Dickson

Debbie Emmerson

Sally Everitt

Virginia Fajt

Marco Fantinati

Erik Fausak

Marisa Ferreira

Myra Forster-van-Hijfte

Ben Garland

Oliver Gilman

Eloi Guarnieri

Sandra Guillen

Simon Hagley

Emily Hall

Jennifer Hamlin

Katherine Hart

Annarita Imperante

Joanne Ireland

Erin Jones

Kenneth Joubert

Claire Kealey

Simone Kirby

Samantha Lindley

Alison Livesey

Loni Loftus

Tafara Mapuvire

Miltiadis Markou

Matthijs Metselaar

Paloma Moreno

Joanna Morris

Kristina Muise

Conor O’Halloran

James Oxley

Carolina Palacios Jimenez

Valentin Parmen

Christopher Parratt

Jonathan Pink

Marc Raffe

Ian Ramsey

Adam Revitt

Clara Rigotti

Emma Robertson

Janet Rodgers

Lynda Rutherford

Ian Self

Shameena Shajira

Bruce Smith

Eva Spada

Thaleia Stathopoulou

Emmelie Stock

Morag Sutherland

Adam Swallow

Emma Tallini

Sarah Tayler

Sumuduni Theminimulle

Ruchita Uttarwar

Nieky van Veggel

Enzo Vettorato

Kate White

Diana Williams

Kirsty Young

Bozena Zaleska

### ORCiD

Peter Cockcroft: https://orcid.org/0000-0003-0090-07060

### Disclaimer

Any opinions expressed in articles and other publication types published in *Veterinary Evidence* are the author's own and do not necessarily reflect the view of the RCVS Knowledge. *Veterinary Evidence* is a resource to help inform, and the content herein should not override the responsibility of the practitioner. Practitioners should also consider factors such as individual clinical expertise and judgement along with patient’s circumstances and owners’ values. Authors are responsible for the accuracy of the content. While the Editor and Publisher believe that all content herein are in accord with current recommendations and practice at the time of publication, they accept no legal responsibility for any errors or omissions, and make no warranty, express or implied, with respect to material contained within. For further information please refer to our Terms of Use.

